# Concisely Constructing S, F Co-Modified MnO Nanoparticles Attached to S, N Co-Doped Carbon Skeleton as a High-Rate Performance Anode Material

**DOI:** 10.3390/molecules29184306

**Published:** 2024-09-11

**Authors:** Dan Zhang, Chunyan Zhang, Zhe Huo, Jia Sun, Guangyin Liu, Xiaodi Liu, Chuang Yu

**Affiliations:** 1 College of Chemistry and Pharmaceutical Engineering, Nanyang Normal University, Nanyang 473061, China; zhangchunyanny@163.com (C.Z.); imkc62@163.com (Z.H.); sj18623839768@163.com (J.S.); liugy13@163.com (G.L.);; 2 State Key Laboratory of Advanced Electromagnetic Engineering and Technology, School of Electrical and Electronic Engineering, Huazhong University of Science and Technology, Wuhan 430074, China

**Keywords:** MnO, carbon, co-doped, anode, lithium-ion batteries, rate performance

## Abstract

The utilization of MnO anodes with high storage capacity is significantly hindered by rapid capacity fading and inadequate rate capability, stemming from substantial volume fluctuations and low electrical conductivity. Crafting a composite comprising sulfur and fluorine co-modified MnO nanoparticles integrated with sulfur and nitrogen co-doped carbon matrices promises enhanced electrochemical performance yet poses formidable obstacles. Here, we present a straightforward synthetic strategy for in situ growth of sulfur and fluorine co-modified MnO nanoparticles onto sulfur and nitrogen co-doped carbon scaffolds. This integration effectively mitigates volume variations and enhances electrical conductivity. As a result, the SF-MnO/SNC composite demonstrates remarkable cycling stability and rate capability when employed as a lithium-ion battery anode. Remarkably, it achieves a high reversible capacity of 975 mAh g^−^¹ after 80 cycles at 0.1 A g^−^¹ and retains a substantial capacity of 498 mAh g^−^¹ even at a high rate of 2.0 A g^−^¹. The concise synthesis method and exceptional rate properties render the SF-MnO/SNC composite a promising anode material for lithium-ion batteries. The strategy of simultaneously doping oxides and carbon will bring new ideas to the research of oxide anodes.

## 1. Introduction

Environmental degradation and global warming have compelled humanity to explore alternative energy sources for replacing traditional fossil fuels. Lithium-ion batteries (LIBs) have emerged as a most prevalent energy source in daily life due to their long lifespan, low operating costs, and minimal environmental impact [1,2,3,4,5]. However, the current use of graphite as the anode material in LIBs poses a limitation: Its relatively low energy density results in prolonged charging times, significantly hindering the rapid adoption of LIBs in electric vehicles [6,7,8,9]. Therefore, the research into high-capacity negative electrode materials is paramount for the swift development and widespread implementation of LIBs in the electric vehicle industry. Indeed, transition-metal oxide materials have garnered significant attention due to their remarkable reversible capacities, which far exceed those of traditional graphite anode material [10,11,12]. Among these materials, MnO stands out as a particularly promising anode candidate for high-performance LIBs. Its high theoretical capacity coupled with its natural abundance, non-toxicity, and low cost make MnO an attractive choice for advancing LIBs technology [13,14,15]. Nevertheless, despite these advantages, MnO faces significant challenges that hinder its practical application. Notably, the material undergoes substantial volume changes during charge-discharge cycles, leading to structural instability and rapid capacity fade. Additionally, MnO exhibits low electrical conductivity, which limits its rate performance and further exacerbates its capacity decay. Overcoming these challenges is crucial for realizing the full potential of MnO as a high-performance anode material for LIBs.

Currently, two primary strategies have been devised to surmount the drawbacks mentioned above. One approach involves reducing the particle size to the nanoscale. The approach would make the diffusion path and storage time of lithium ions short while also minimizing volume changes [16,17,18,19]. The second strategy entails integrating MnO with conductive carbon. This not only enhances the electrical conductivity of the final composite but also accommodates volume changes during cycling processes, thereby improving rate capability and cycling stability [20,21,22]. Apparently, it is logical to assume that the synergistic effect of these two strategies, that is, preparing a composite of MnO nanoparticles and carbon, could lead to superior electrochemical performance. Indeed, many research studies on MnO anodes had similar trains of thought [23,24,25]. However, research on how to further enhance the electrochemical performance based on these foundations remains relatively scarce. 

Through a thorough analysis of a substantial body of literature on anode materials, we think element doping to modify MnO and carbon at the same time can further elevate the electrochemical performance. Sulfur doping of metal oxides has been shown to enhance the reversible capacity of the material [26,27,28]. This is attributed to the modification of the crystal structure and electronic properties of manganese oxide, facilitating better lithium-ion insertion and extraction processes. Fluorine doping, on the other hand, has been proven to improve the rate capability of metal oxides [29,30,31]. Fluorine atoms can stabilize the structure and increase the conductivity of the material, allowing for faster lithium-ion diffusion. Additionally, both sulfur and nitrogen are introduced into the carbon matrix, which can significantly boost the conductivity of carbon, leading to improved overall electrochemical performance [32,33,34]. Sulfur doping can increase the charge carrier density, while nitrogen doping can introduce additional active sites and improve the wettability of the carbon surface. Both these doping strategies offer promising avenues for further enhancing the performance of MnO-based materials for use in lithium-ion batteries, complementing the strategies of nanosizing and carbon composite formation. However, conveniently synthesizing the composite of S, F co-doped MnO nanoparticles and S, N co-doped carbon (donated as SF-MnO/SNC) still poses huge challenges. Therefore, there is a pressing need to develop a concise synthesis route for the preparation of SF-MnO/SNC and further explore their performance as anodes for LIBs.

In this research, we devised an efficient approach to synthesize a composite material comprising S, F co-doped MnO nanoparticles embedded within S, N co-doped carbon. This composite (SF-MnO/SNC) effectively mitigates the challenge of volume expansion and significantly enhances electrochemical reaction kinetics. When employed as the anode material in LIBs, the SF-MnO/SNC composite exhibits exceptional electrochemical performance.

## 2. Results and Discussion

Our synthesis methodology primarily encompasses two key stages: the formation of the sol and the subsequent calcination process. Polyacrylamide was selected due to its excellent coordination with manganese ions, enabling the formation of a high-quality gel. Turning to the calcination process, polyacrylic amide serves a dual purpose, acting as both a carbon source and a nitrogen source. Furthermore, NH_4_F contributes as a fluorine source and provides an additional nitrogen source. Similarly, (NH_4_)_2_SO_4_ acts as a sulfur source and provides an additional nitrogen source. Lastly, sodium chloride plays a pivotal role as a templating agent, facilitating the creation of porous structures. The synthesis technology revolves around freeze drying and calcination, which are straightforward, user-friendly, and conducive to industrial-scale production.

**Figure 1:**
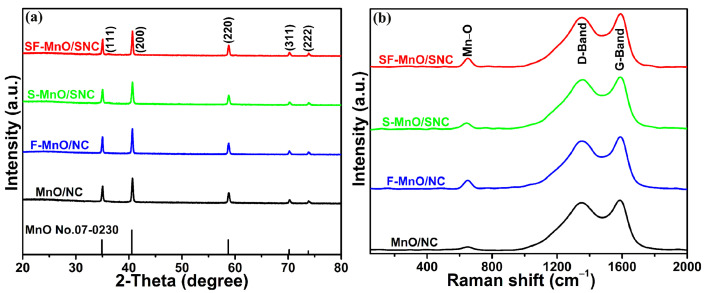
XRD patterns (**a**) and Raman spectra (**b**) of SF-MnO/SNC, S-MnO/SNC, F-MnO/NC, and MnO/NC composites.

The crystalline architectures of the four prepared specimens were scrutinized using XRD analysis. As depicted in Figure 1a, the diffraction peaks align with the (111), (200), (220), (311), and (222) diffraction planes, respectively. The diffraction patterns of SF-MnO/SNC, S-MnO/SNC, F-MnO/NC, and MnO/NC products conform to the standard MnO phase (JCPDS No. 07-0230). Based on Debye–Scherrer formula, it can be estimated that the size of the MnO nanoparticles for the SF-MnO/SNC sample is 36 nanometers. To delve deeper into the subtle structural nuances of these samples, the Raman spectra are presented in Figure 1b. Two prominent peaks, observed within the ranges of 1345~1360 cm^−1^ and 1585~1595 cm^−1^, in all four samples, correspond to the D-band and G-band of carbon, respectively [35,36]. Additionally, the peaks located at 640~655 cm^−1^ in all four samples are attributed to the stretching vibration of the Mn−O bond in manganese oxide, which is in line with the previous literature [37,38]. These findings indicate that our products are indeed composites of carbon and manganese oxide. 

**Figure 2:**
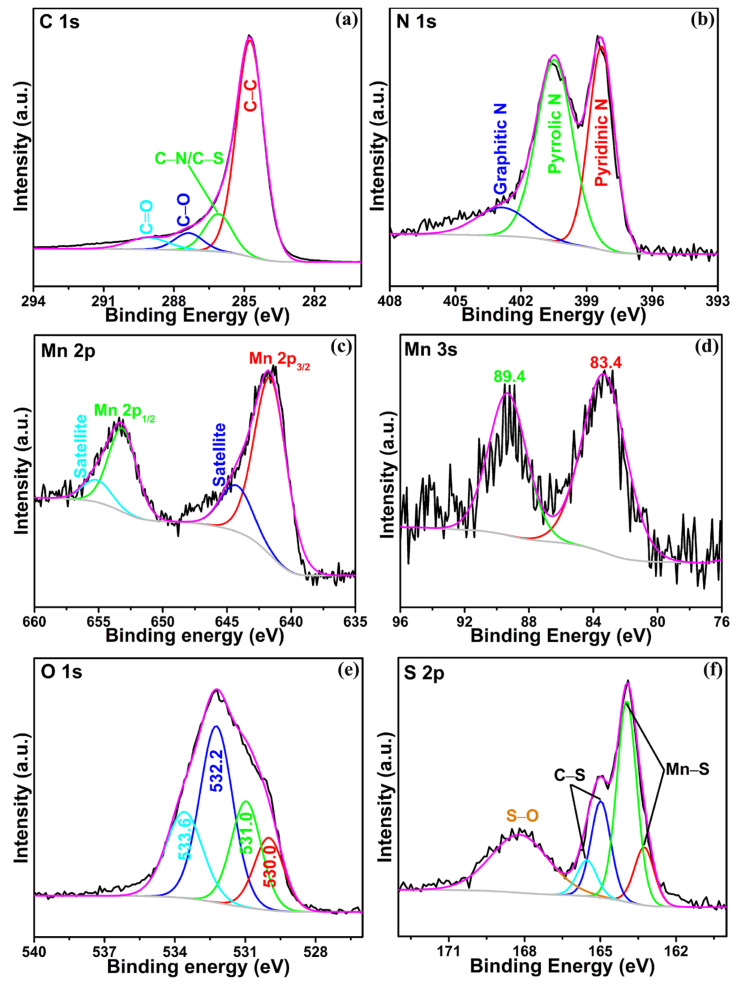
XPS spectra of the C 1s region (**a**), N 1s region (**b**), Mn 2p region (**c**), Mn 3s region (**d**), O 1s region (**e**), and S 1s region (**f**) for the SF-MnO/SNC composite.

X-ray photoelectron spectroscopy (XPS) was utilized to further elucidate the chemical state of the SF-MnO/SNC composite. The high-resolution XPS spectra for C 1s, N 1s, Mn 2p, Mn 3s, O 1s, S 2p, and F 1s are presented in Figure 2a–f and Figure S1. The C 1s spectrum (Figure 2a) reveals four deconvoluted peaks at 284.8, 286.1, 287.4, and 289.1 eV, corresponding to C−C, C−N/C−S, C−O, and C=O bonds, respectively [28]. The presence of C−N/C−S bonds signifies the successful doping of nitrogen and sulfur into the carbon matrix of the SF-MnO/SNC composite. The N 1s XPS spectrum (Figure 2b) exhibits three distinct peaks centered at 398.3, 400.5, and 402.9 eV, attributed to pyridinic N, pyrrolic N, and graphitic N, respectively [39,40]. This finding reinforces the notion of nitrogen doping in the carbon of the SF-MnO/SNC composite, aligning with the C 1s results. The doping amount of N for carbon was estimated to be ~6.5 at% through XPS analysis. Nitrogen doping introduces additional active sites within the carbon structure and improves the conductivity. The Mn 2p spectrum (Figure 2c) displays two primary peaks positioned at 641.7 and 653.3 eV, indicative of the 2p_3/2_ and 2p_1/2_ spin-orbit split of Mn^2+^, confirming the presence of Mn^2+^ in the composite. Satellite peaks at 644.4 and 655.3 eV are also observed, corresponding to the 2p_3/2_ and 2p_1/2_ shake-up features. These findings are consistent with previous reports on MnO materials [41,42]. To further probe the oxidation state of Mn, the Mn 3s region was analyzed. The spacing between Mn 3s peaks varies according to the oxidation state of Mn. In this study, the Mn 3s spectrum (Figure 2d) exhibits two peaks, indicative of electron coupling. Notably, an energy separation of 6.0 eV in the spectrum corresponds to the Mn^2+^ state [43], confirming the presence of MnO in the composite, in agreement with the Mn 2p results. As depicted in Figure 2e, the deconvoluted O 1s spectrum reveals four distinct peaks. The peak centered at 530.0 eV corresponds to the Mn–O bond [44]. The peak situated at 531.0 eV is probably attributed to the Mn–O–C bond [45]. The presence of Mn–O–C signifies a clear chemical interaction between the S, F co-doped MnO and S, N co-doped carbon. Two further peaks at 532.2 eV and 533.6 eV align with adsorbed hydroxyl groups [46]. Notably, the formation of Mn–O–C bonds can significantly enhance charge transfer rates and promote electrochemical reversibility. Turning to Figure 2f, it presents the high-resolution XPS spectrum of S 2p. Two peaks at 163.2 and 164.0 eV are indicative of Mn–S bonds [47]. Additionally, the peaks at 165.0 eV and 165.5 eV correspond to C–S–C bonds within the carbon matrix [48]. Moreover, the broad peak centered at 168.2 eV is attributed to the formation of S–O bonds, arising from oxidation of the sample [49]. These findings reinforce the successful doping of sulfur into both MnO and carbon. The doping amount of S for MnO nanoparticles was estimated to be ~7.9 at% through XPS analysis. Regarding the F 1s spectrum (Figure S1), it displays a single peak at 687.0 eV, which stems from the Mn–F bond [50]. The finding corroborates the successful doping of fluorine into MnO. The doping amount of F for MnO nanoparticles was estimated to be ~2.9 at% through XPS analysis. The XPS results suggest that our products are indeed composites of S, N co-doped carbon and S, F co-doped manganese oxide. 

**Figure 3:**
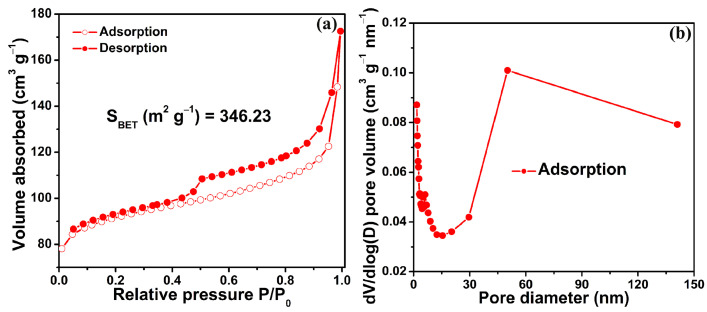
N_2_ adsorption–desorption isotherm (**a**) and pore size distribution (**b**) of the SF-MnO/SNC composite.

To gain profound insights into the porous architecture of the SF-MnO/SNC composite, N_2_ adsorption/desorption analysis was employed. As depicted in Figure 3a, the N_2_ sorption isotherm of the SF-MnO/SNC composite exhibits a Type IV profile, indicating a significant abundance of pores within the material [51]. This composite boasts a substantial BET-specific surface area of 346.23 m^2^ g^−1^, accompanied by a pore volume of 0.27 cm^3^ g^−1^. Figure 3b illustrates the pore size distribution, revealing a predominance of mesopores ranging from 2 to 8 nm, with macropores centered around 50 nm. The formation of this porous structure in the SF-MnO/SNC composite can be attributed primarily to the decomposition of polyacrylic amide during calcination and the subsequent removal of the sodium chloride template through washing procedures. 

**Figure 4:**
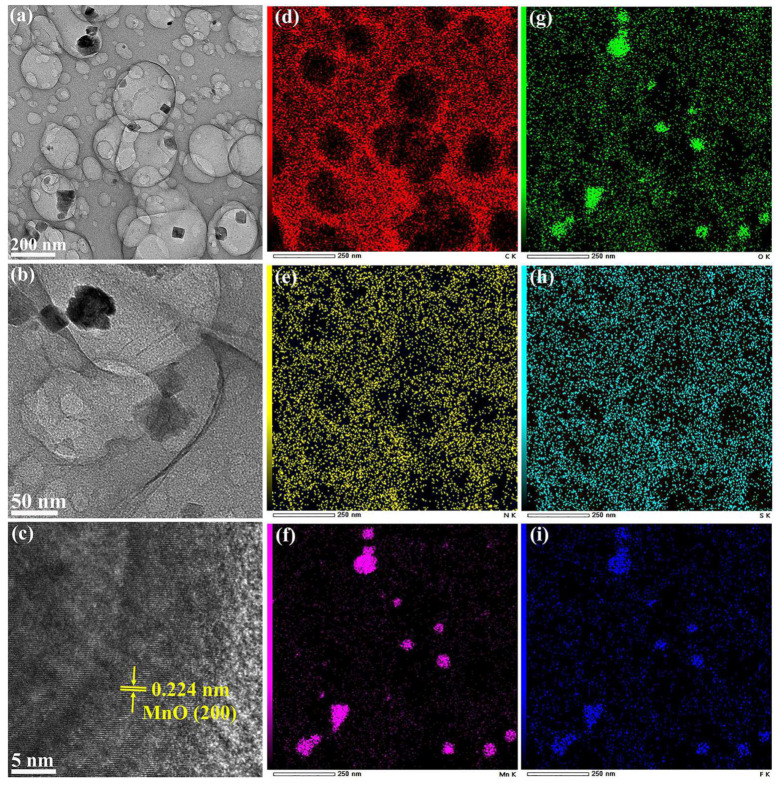
TEM images (**a**,**b**), HRTEM image (**c**) and EDS maps of elemental C (**d**), N (**e**), Mn (**f**), O (**g**), S (**h**), and F (**i**) for SF-MnO/SNC composite.

Figure 4a,b show the TEM images of the SF-MnO/SNC composite, clearly revealing that the majority of S, F co-doped MnO nanoparticles exhibit an irregular cubic block morphology, intimately adhered to the S, N co-doped carbon framework. Notably, the carbon framework displays an abundance of pore structures, corroborating the BET analysis findings. Figure S2 shows the particle size distribution of the SF-MnO/SNC composite, from which we can know that the average particle size of MnO nanoparticles is 43 nanometers. This result is very close to the data calculated by the Debye–Scherrer formula. Figure 4c presents a HRTEM image, where the discernible lattice fringe spacing of 0.224 nm aligns with the (200) plane of MnO. Additionally, Figure S3 and Figure 4d–i display the corresponding HAADF image and elemental mapping of the SF-MnO/SNC composite. The concentrated clustering of Mn, O, and F elements within the nanoparticle regions confirms the successful incorporation of F into MnO. The uniform distribution of C and N elements within the carbon framework validates N-doping into the carbon matrix. Similarly, the uniform presence of S across both MnO nanoparticles and the carbon framework attests to the successful doping of S into both MnO and carbon. These elemental mapping results reinforce that our products are indeed composites composed of S, N co-doped carbon and S, F co-doped manganese oxide, in harmony with the XPS outcomes.

The TEM image presented in Figure S4 for the S-MnO/SNC composite conclusively verifies the growth of sulfur-doped MnO nanoparticles onto sulfur and nitrogen co-doped porous carbon scaffold. Figure S5 subsequently showcases the corresponding HAADF image along with elemental mapping, revealing a clear congregation of Mn and O elements at the nanoparticle sites, confirming their identity. The uniform distribution of C and N elements throughout the carbon framework attests to the successful nitrogen doping. Additionally, the ubiquitous presence of sulfur across both MnO nanoparticles and the carbon framework underscores sulfur’s incorporation into both MnO and carbon, reinforcing the composite’s composition as S, N co-doped carbon integrated with S-doped manganese oxide. Similarly, the TEM image in Figure S6 for the F-MnO/NC composite verifies the growth of fluorine-doped MnO nanoparticles on an N-doped porous carbon structure. Figure S7 displays the accompanying HAADF image and elemental mapping, demonstrating the accumulation of Mn, O, and F elements at the nanoparticle locations, indicative of fluorine doping in MnO. The uniformity of C and N elements within the carbon framework confirms nitrogen doping. These elemental mapping results further validate the composition of the F-MnO/NC composite as a blend of N-doped carbon and fluorine-doped manganese oxide. Lastly, the TEM image in Figure S8a for the MnO/NC composite verifies the presence of MnO nanoparticles grown on an N-doped porous carbon framework. Figure S8b–f present the corresponding HAADF image and elemental mapping, showcasing the clustering of Mn and O elements at the nanoparticle sites. The uniform distribution of C and N elements within the carbon framework verifies nitrogen doping. These elemental mapping outcomes conclusively indicate that the MnO/NC composite comprises N-doped carbon integrated with manganese oxide. The particle size of the MnO nanoparticles for MnO/NC composite is about 200 nm. The particle size of the MnO nanoparticles for S-MnO/SNC composite is about 150~180 nm. The particle size of the MnO nanoparticles for F-MnO/NC composite is about 80~150 nm. It is obvious that doping with sulfur or fluorine alone will result in a decrease in particle size, while doping with both sulfur and fluorine will result in a smaller particle size. This phenomenon is consistent with the literature that doping can reduce particle size.

**Figure 5:**
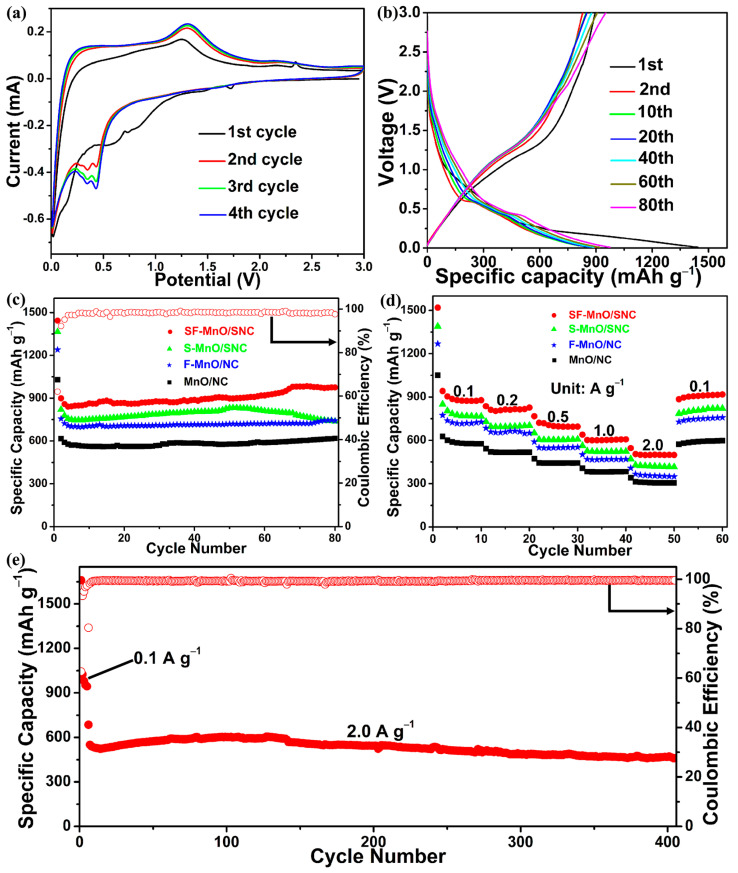
(**a**) CV curves recorded at a scan rate of 0.2 mV s^−1^ and (**b**) discharge–charge potential profiles of the SF-MnO/SNC anode material at a current density of 0.1 A g^−1^. (**c**) Cycling stability and Coulombic efficiency of the SF-MnO/SNC anode, in comparison with S-MnO/SNC, F-MnO/NC, and MnO/NC anodes, all tested at 0.1 A g^−1^. (**d**) Rate performance evaluation of the SF-MnO/SNC, S-MnO/SNC, F-MnO/NC, and MnO/NC anode materials. (**e**) Cycling stability and Coulombic efficiency of the SF-MnO/SNC anode after activation, tested under a high current density of 2.0 A g^−1^.

The electrochemical properties of SF-MnO/SNC, S-MnO/SNC, F-MnO/NC, and MnO/NC were investigated as potential anode materials for LIBs. Figure 5a presents the initial four cyclic voltammetry (CV) curves of the SF-MnO/SNC anode at a scan rate of 0.2 mV s^−^¹. During the initial discharge cycle, a peak observed at 1.72 V disappears in subsequent discharges, possibly attributed to the formation of a solid electrolyte interphase (SEI) film [52]. The broad reduction peaks in the range of 0.4 to 0.8 V are likely due to the insertion of lithium ions into MnO and the formation of Mn and Li₂O [53]. In subsequent discharge cycles, these reduction peaks shift to a lower potential range of 0.2 to 0.5 V, primarily because of changes in the electrode’s microstructure and reaction kinetics after the first cycle [54]. During the initial charge cycle, the SF-MnO/SNC anode exhibits a prominent oxidation peak at 1.26 V, corresponding to the release of lithium ions from Li₂O and the conversion of Mn to MnO [55]. Notably, a weaker oxidation peak at 2.34 V is also observed: a phenomenon reported elsewhere, stemming from the formation of higher oxidation states of manganese (Mn³⁺ or Mn⁴⁺) and enhanced conductivity facilitated by heteroatom-doped carbon, thereby contributing additional capacity to the electrode material [56]. During subsequent charge cycles, the oxidation peak shifts to a slightly higher potential of 1.31 V. Comparing the third and fourth cyclic curves, minimal changes are evident, underscoring the excellent electrochemical reversibility of the SF-MnO/SNC anode.

Figure 5b presents a diverse range of discharging–charging potential profiles spanning from 0.01 to 3 V for the SF-MnO/SNC anode material under a current density of 0.1 A g^−^¹. Notably, the initial discharge and charge capacities for the SF-MnO/SNC anode are 1444 and 896 mAh g^−^¹, respectively, with an initial Coulombic efficiency of 62%. The capacity loss observed during the initial cycle is probably because of the formation of a SEI layer. During discharge, the voltage plateau around 0.5 V signifies the reduction of MnO to manganese, whereas during charging, the voltage plateau near 1.3 V represents the oxidation of Mn back to MnO. These findings are in harmony with the CV results. Following the initial cycle, the potential profiles exhibit minimal variations, indicative of exceptional electrochemical reversibility for the SF-MnO/SNC anode.

Figure 5c presents the cycling performance of SF-MnO/SNC, S-MnO/SNC, F-MnO/NC, and MnO/NC anodes over 80 cycles at a current density of 0.1 A g^−^¹. Following 80 cycles, the SF-MnO/SNC anode demonstrates a remarkable discharge capacity of 975 mAh g^−^¹, which is 2.6 times of the theoretical capacity for commercial graphite anodes. The average Coulombic efficiency across all 80 cycles is approximately 98%, suggesting excellent electrochemical stability for the SF-MnO/SNC anode. Notably, a slight increase in capacity is observed during cycling, which, according to extensive literature, can be attributed to particle pulverization, resulting in smaller particle sizes and the emergence of new electrochemical active sites [57]. In contrast, the MnO/NC electrode exhibits the lowest discharge capacity of 618 mAh g^−^¹ after 80 cycles. The S-MnO/SNC and F-MnO/NC electrodes, with discharge capacities of 738 and 742 mAh g^−^¹, respectively, outperform the MnO/NC electrode, demonstrating that the incorporation of sulfur and fluorine enhances reversible capacity. Furthermore, the F-MnO/NC electrode exhibits superior cyclic stability compared to S-MnO/SNC, indicating that fluorine also contributes to improved electrode stability. The SF-MnO/SNC electrode, with the best overall electrochemical performance, underscores the advantage of simultaneously incorporating sulfur and fluorine for enhancing electrochemical properties.

Figure 5d showcases the discharge capacities achieved at varying current densities ranging from 0.1 to 2.0 A g^−^¹. Notably, the SF-MnO/SNC anode demonstrates the superior rate capability, delivering discharge capacities of 878, 827, 695, 607, and 498 mAh g^−^¹ at the 10th cycle as the current density escalates from 0.1 to 2.0 A g^−^¹. Remarkably, even when the current density is reverted to 0.1 A g^−^¹, the discharge capacity recovers to 918 mAh g^−^¹, proving that electrode materials have good adaptability to changes in current density. In contrast, the S-MnO/SNC electrode exhibits lower discharge capacities than SF-MnO/SNC anode, achieving 702, 607, 523, and 415 mAh g^−^¹ at the 10th cycle under 0.2, 0.5, 1.0, and 2.0 A g^−^¹, respectively. Similarly, the performance of the F-MnO/NC electrode is also worse than the SF-MnO/SNC anode, with discharge capacities of 650, 552, 469, and 349 mAh g^−^¹ under the same conditions. Among these electrodes, the MnO/NC electrode displays the poorest performance, with discharge capacities of 518, 443, 382, and 304 mAh g^−^¹ at the 10th cycle under the same conditions. Figure 5e presents the cycling stability of the SF-MnO/SNC anode at 2.0 A g^−^¹ for 400 cycles, following an initial activation period of five cycles. This graph underscores the exceptional cyclic durability of the SF-MnO/SNC electrode, which maintains a substantial discharge capacity of 460 mAh g^−^¹ even after 400 cycles at 2.0 A g^−^¹, while boasting an average Coulombic efficiency of 99% throughout the entire cycling process.

**Figure 6:**
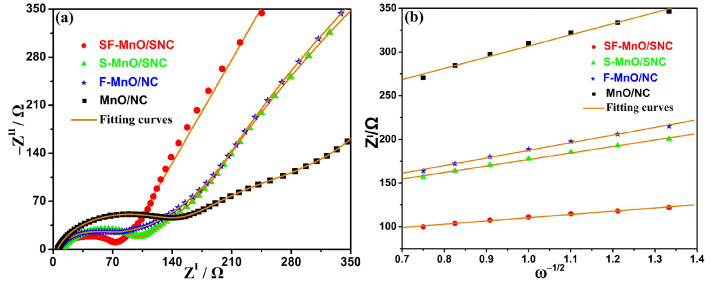
(**a**) Electrochemical impedance spectra after five cycles at a current density of 0.1 A g^−^¹ and (**b**) data fitting outcomes of plots for various anodes, including SF-MnO/SNC, S-MnO/SNC, F-MnO/NC, and MnO/NC.

The electrochemical impedance spectra (EIS) for the SF-MnO/SNC, S-MnO/SNC, F-MnO/NC, and MnO/NC negative electrodes after five cycles are presented in Figure 6a. These spectra were fitted using the equivalent circuit depicted in Figure S9, and the corresponding fitted data are provided in Table S1. Specifically, the SEI film resistances (R_f_) for the SF-MnO/SNC, S-MnO/SNC, F-MnO/NC, and MnO/NC electrodes are 27, 41, 36, and 62 Ω, respectively. Similarly, the charge transfer resistances (R_ct_) are 35, 54, 51, and 70 Ω. The notably lower R_f_ and R_ct_ values observed for S-MnO/SNC and F-MnO/NC compared to MnO/NC underscore the effectiveness of sulfur and fluorine doping in individually reducing electrochemical impedance. Furthermore, the lowest R_f_ and R_ct_ values achieved by SF-MnO/SNC indicate a synergistic effect from simultaneous sulfur and fluorine doping, leading to optimal electrochemical impedance, which facilitates rapid lithium ion and electron transport, thereby enhancing electrochemical performance [58]. To quantify this further, we calculated the lithium-ion diffusion coefficients (D_Li_^+^) for the SF-MnO/SNC, S-MnO/SNC, F-MnO/NC, and MnO/NC electrodes using Equation (1) [59]:

D_Li_^+^ = (R^2^T^2^)/(2A^2^n^4^F^4^ C^2^σ^2^) (1)

In this equation, R, T, A, n, F, and C stand for the gas constant, Kelvin temperature, electrode surface area, number of electrons involved in the electrochemical reaction, Faraday constant, and lithium-ion concentration in the electrode, respectively. The value of *σ* is the gradient fitted from the Z^I^ versus ω^−1/2^ plot in the Warburg region. As shown in Figure 6b, the σ values for SF-MnO/SNC, S-MnO/SNC, F-MnO/NC, and MnO/NC are 37.2, 74.4, 87.4, and 127.9, respectively. Applying Equation (1), we obtain D_Li_^+^ values of 1.4 × 10^−16^, 3.5 × 10^−17^, 2.5 × 10^−17^, and 1.1 × 10^−17^ cm^2^ s^−1^ for the respective electrodes. Evidently, the higher D_Li_^+^ values for S-MnO/SNC and F-MnO/NC compared to MnO/NC confirm that individual sulfur and fluorine doping accelerates lithium-ion diffusion, underscoring the benefits of these doping strategies. The exceptional D_Li_^+^ value exhibited by SF-MnO/SNC underscores the synergy attained through concurrent sulfur and fluorine doping, resulting in unparalleled electrochemical reaction kinetics and best rate performance. As a result, the SF-MnO/SNC anode demonstrates remarkable electrochemical performance, standing out as the optimal choice. 

**Figure 7:**
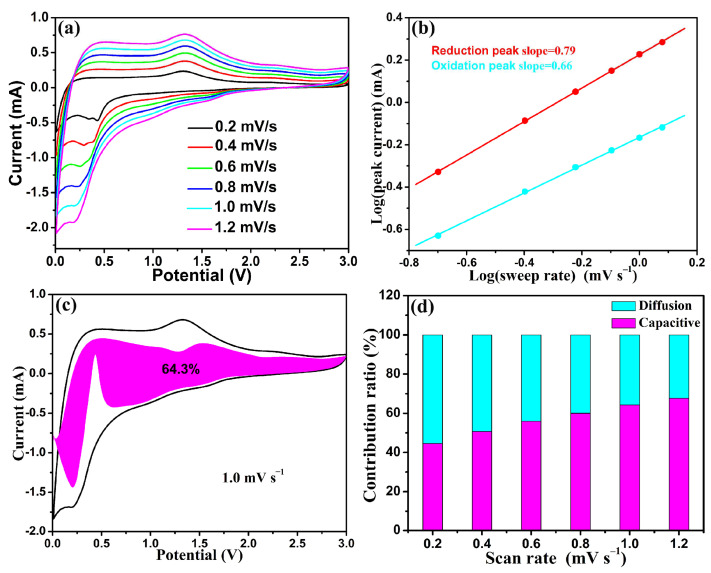
(**a**) CV curves at different sweep rates, (**b**) a plot of log (peak current) versus log (sweep rate) at specific current peaks, (**c**) a CV profile highlighting the capacitive contribution at a sweep rate of 1.0 mV s^−^¹, and (**d**) a normalized comparison of diffusion and capacitive ratios across various sweep rates for the SF-MnO/SNC anode.

To further delve into the underlying reasons for the exceptional electrochemical performance of the SF-MnO/SNC anode, CV curves were recorded at various scanning rates ranging from 0.2 to 1.2 mV s^−^¹. As evident from Figure 7a, minimal variations in the electrode’s configuration were observed across the varying scanning rates, suggesting electrode stability. Typically, the relationship between current density (*i*) and scanning speed (*v*) adheres to Equation (2) [60]:

*i* = a*v*^b^ (2)

Utilizing Equation (2), the gradient of the log *v*–log *i* plot yields the value of b. When 0.5 < b < 1, it signifies a hybrid process encompassing both capacitive-dominated and ionic diffusion-controlled mechanisms [61]. As depicted in Figure 7b, the b values for the cathodic and anodic peaks are 0.79 and 0.66, respectively, confirming that the lithium-ion storage in the SF-MnO/SNC anode proceeds through a concurrent combination of capacitive and ionic diffusion-controlled processes. Furthermore, the contribution ratios of these two mechanisms can be quantitatively determined using Equation (3) [62]:

*i* = k_1_*v* + k_2_*v*^1/2^ (3)

Figure 7c demonstrates that at a scan rate of 1.0 mV s^−1^, 64.3% of the capacitive contribution stems from pseudo-capacitive processes. Additionally, as illustrated in Figure 7d, with the increase in scan rates from 0.2 to 1.2 mV s^−1^ (0.2, 0.4, 0.6, 0.8, 1.0, and 1.2 mV s^−1^), the capacitance contribution rates gradually rise to 44.6%, 50.7%, 55.9%, 60.0%, 64.3%, and 67.7%, respectively. These findings confirm that the capacitive-dominated process dominates the SF-MnO/SNC anode when the scan rates exceed 0.4 mV s^−1^. Under high scan rates, the capacitive-dominated process facilitates rapid lithium-ion storage, thereby enhancing the exceptional rate performance of the SF-MnO/SNC negative electrode.

## 3. Experimental Section

### 3.1. Synthesis of SF-MnO/SNC Composite

The SF-MnO/SNC sample was synthesized through the subsequent steps. Initially, 0.8 g of polyacrylamide, 0.8 g of NaCl, 0.04 g of ammonium sulfate ((NH_4_)_2_SO_4_), and 0.04 g of ammonium fluoride (NH_4_F) were combined in 40 mL of distilled water and agitated for 10 min to produce Solution 1. Subsequently, 1.8 g of manganese gluconate was dissolved in 20 mL of distilled water and stirred for 10 min to form Solution 2. Next, Solution 2 was introduced dropwise into Solution 1, followed by stirring for 60 min. The resulting gel was subjected to cold treatment at −18 °C for 12 h, thereafter undergoing freeze drying for 36 h. The obtained powders were then heated to 650 °C for 120 min in an inert atmosphere. Finally, the materials were rinsed with distilled water, filtered under vacuum, and dried at 60 °C to collect the SF-MnO/SNC sample. For comparison purposes, the S-MnO/SNC sample was prepared using the same procedure but excluding NH_4_F while keeping all other conditions unchanged. Similarly, the F-MnO/NC sample was synthesized without (NH_4_)_2_SO_4_, keeping the rest of the synthesis conditions constant. Lastly, the MnO/NC sample was obtained by omitting both NH_4_F and (NH_4_)_2_SO_4_, with all other synthesis parameters remaining the same. 

### 3.2. Materials Characterization

The XRD patterns were acquired utilizing a Rigaku SmartLab SE diffractometer (Rigaku, Tokyo, Japan). The transmission electron microscopy (TEM) imagery and energy-dispersive spectroscopy (EDS) outcomes were derived from a JEOL JEM-F200 microscope (JEOL, Tokyo, Japan). Nitrogen adsorption-desorption isotherms of the sample were precisely measured on a Micromeritics ASAP 2460 analyzer (Micromeritics, Norcross, GA, USA). The Raman spectroscopic analysis was conducted on a Horiba LabRAM HR Evolution system (Horiba, Kyoto, Japan). For XPS, data were collected via a Thermo Scientific K-Alpha instrument (Thermo Scientific, Waltham, MA, USA). The electrochemical assessments were systematically performed using a LANHE Battery Test System (LANHE, Wuhan, China) in conjunction with an electrochemical workstation (Chenhua, Shanghai, China), specifically the CHI660C model. Additional details pertaining to electrode fabrication, battery construction, and the execution of electrochemical tests are provided in the Supporting Information. 

## 4. Conclusions

To summarize, a concise method was employed to successfully synthesize a composite consisting of S, F co-doped MnO nanoparticles integrated with an S, N co-doped porous carbon framework. The battery performance assessments affirmed that the SF-MnO/SNC anode demonstrates an impressive operational lifespan and superior rate capability. Notably, even after enduring 400 cycles at a current density of 2.0 A g^−^¹, the SF-MnO/SNC anode maintained a remarkable capacity of 460 mAh g^−^¹. This exceptional rate performance is likely attributed to the synergistic effect of the S, F co-doped MnO nanoparticles paired with the S, N co-doped porous carbon framework, which significantly mitigates volume expansion while dramatically enhancing the lithium-ion diffusion rate and conductivity. This research offers invaluable insights into the design of high-performance metal oxide-based anode materials for LIBs.

## Data Availability

Under reasonable request, the data of this work are available from the corresponding author. The data are not publicly available due to institutional restriction.

## Supplementary Materials

The following supporting information can be downloaded at: https://www.mdpi.com/article/10.3390/molecules29184306/s1. Figure S1: XPS spectrum of the F 1s region for SF-MnO/SNC composite; Figure S2: Nanoparticle size distribution diagram for SF-MnO/SNC composite; Figure S3: HAADF image of the elemental mapping for SF-MnO/SNC composite; Figure S4: TEM image of S-MnO/SNC composite; Figure S5: HAADF image of the elemental mapping and EDS maps of S-MnO/SNC composite; Figure S6: TEM image of F-MnO/NC composite; Figure S7: HAADF image of the elemental mapping and EDS maps of F-MnO/NC composite; Figure S8: TEM image, HAADF image of the elemental mapping and EDS maps of MnO/NC composite; Figure S9: Equivalent circuit; Table S1: EIS parameters for the SF-MnO/SNC, S-MnO/SNC, F-MnO/NC, and MnO/NC electrodes derived through the fitting of experimental data to an equivalent circuit model.

## References

[1] Liu J.-H., Wang P., Gao Z., Li X., Cui W., Li R., Ramakrishna S., Zhang J., Long Y.-Z. (2024). Review on electrospinning anode and separators for lithium ion batteries. Renew. Sust. Energ. Rev..

[2] Shi J., Jiang K., Fan Y., Zhao L., Cheng Z., Yu P., Peng J., Wan M. (2024). Advancing Metallic Lithium Anodes: A Review of Interface Design, Electrolyte Innovation, and Performance Enhancement Strategies. Molecules.

[3] Wei C., Liu C., Xiao Y., Wu Z., Luo Q., Jiang Z., Wang Z., Zhang L., Cheng S., Yu C. (2024). SnF_2_-induced multifunctional interface-stabilized Li_5.5_PS_4.5_Cl_1.5_-based all-solid-state lithium metal batteries. Adv. Funct. Mater..

[4] Ding X., Zhou Q., Li X., Xiong X. (2024). Fast-charging anodes for lithium ion batteries: Progress and challenges. Chem. Commun..

[5] Wang Z., Du Z., Wang L., He G., Parkin I.P., Zhang Y., Yue Y. (2024). Disordered materials for high-performance lithium-ion batteries: A review. Nano Energy.

[6] Wang R., Wang L., Liu R., Li X., Wu Y., Ran F. (2024). “Fast-Charging” Anode Materials for Lithium-Ion Batteries from Perspective of Ion Diffusion in Crystal Structure. ACS Nano.

[7] Xing B., Shi F., Jin Z., Zeng H., Qu X., Huang G., Zhang C., Xu Y., Chen Z., Lu J. (2024). A facile ice-templating-induced puzzle coupled with carbonization strategy for kilogram-level production of porous carbon nanosheets as high-capacity anode for lithium-ion batteries. Carbon Energy.

[8] Liu H., Wang S., Zhao J., Zhang B., Liu L., Bao R., Jing Z. (2024). Sn-based anode materials for lithium-ion batteries: From mechanism to modification. J. Energy Storage.

[9] Shen D., Jia M., Li M., Fu X., Liu Y., Dong W., Yang S. (2023). High Coulomb Efficiency Sn–Co Alloy/rGO Composite Anode Material for Li–ion Battery with Long Cycle–Life. Molecules.

[10] Azad A.K., Abdalla A.M., Kumarasinghe P.I.I., Nourean S., Azad A.T., Ma J., Jiang C., Dawood M.M.K., Wei B., Patabendige C.N.K. (2024). Developments and key challenges in micro/nanostructured binary transition metal oxides for lithium-ion battery anodes. J. Energy Storage.

[11] Xu H., Gao C., Cheng Z., Kong L., Li W., Dong X., Lin J. (2023). Metal oxyacid salts-confined pyrolysis towards hierarchical porous metal oxide@carbon (MO@C) composites as lithium-ion battery anodes. Nano Res..

[12] Li X., Guo Y., Gao T., Liu H., Chen C., Li J., Xiao D. (2023). Rational design and construction of iron oxide and titanium carbide MXene hierarchical structure with promoted energy storage properties for flexible battery. J. Colloid Interf. Sci..

[13] Liu R., Zhao X., Zhao H., Liang L., Zhao S., Zhang Y. (2023). Carbon-Coated MnO Quantum Dot-Decorated Three-Dimensional Graphene Aerogel Composite for High-Performance Lithium-Ion Batteries. Energy Fuels.

[14] Lin J., Peng Y., Reddy R.C.K., Zeb A., Lin X., Sun Y.-H. (2023). Carbon-encapsulated anionic-defective MnO/Ni open microcages: A hierarchical stress-release engineering for superior lithium storage. Carbon Energy.

[15] He C., Li J., Zhao X., Peng X., Lin X., Ke Y., Xiao X., Zuo X., Nan J. (2023). In situ anchoring of MnO nanoparticles into three-dimensional nitrogen-doped porous carbon framework as a stable anode for high-performance lithium storage. Appl. Surf. Sci..

[16] Huang S., Xu G., Wei X., Yang L. (2024). Quasi-spherical N-doped Carbon aggregates embedded with massive MnO quantum dots as High-performance Anodes for Li ion batteries. Scripta Mater..

[17] Chen P., Zhou W., Xiao Z., Li S., Chen H., Wang Y., Wang Z., Xi W., Xia X., Xie S. (2020). In situ anchoring MnO nanoparticles on self-supported 3D interconnected graphene scroll framework: A fast kinetics boosted ultrahigh-rate anode for Li-ion capacitor. Energy Storage Mater..

[18] Dong S., Geng H., Yue W., Zheng S., Pang X., Liang J., An G., Li W., Wang B., Lu C. (2023). Single-Shell Multiple-Core MnO@C Hollow Carbon Nanospheres forLow-Temperature Lithium Storage. ACS Appl. Energy Mater..

[19] Zhou F., Li S., Han K., Li Y., Liu Y.-N. (2021). Polymerization inspired synthesis of MnO@carbon nanowires with long cycling stability for lithium ion battery anodes: Growth mechanism and electrochemical performance. Dalton Trans..

[20] Hong Y., Hu Q., Dong H., Li J., Tang Z., Wang X., Ouyang J., Liu T. (2022). N-doped carbon coated porous hierarchical MnO microspheres as superior additive-free anode materials for lithium-ion batteries. Scr. Mater..

[21] Lin J., Zeng C., Lin X., Xu C., Xu X., Luo Y. (2021). Metal−Organic Framework-Derived Hierarchical MnO/Co with Oxygen Vacancies toward Elevated-Temperature Li-Ion Battery. ACS Nano.

[22] Chen J., Yang K., Yu H., Shah T., Zhang Q., Zhang B. (2020). Highly monodisperse dumbbell-like yolk-shell manganese monoxide/carbon microspheres for lithium storage and their lithiation evolution. Carbon.

[23] Liu R., Zhao X., Liang L., Li R., Fan P., Li J., Zhao H. (2023). Double-network aerogel-derived spongy 3D porous MnO/C composite for lithium-ion batteries with increasing capacity. J. Alloys Compd..

[24] Hou J., Guo Y., Zhang Y., Li J., Xu Y., Fang Z., Yang J., Wu M. (2022). Facile green and sustainable synthesis of MnO@rGO as electrochemically stable anode for lithium-ion batteries. Mater. Lett..

[25] Huang S., Li H., Xu G., Liu X., Zhang Q., Yang L., Cao J., Wei X. (2020). Porous N-doped carbon sheets wrapped MnO in 3D carbon networks as high-performance anode for Li-ion batteries. Electrochim. Acta.

[26] Yao W., Qiu W., Xu Z., Xu J., Luo J., Wen Y. (2018). Two-dimensional sulfur-doped Mn_3_O_4_ quantum dots/reduced graphene oxide nanosheets as high-rate anode materials for lithium storage. Ceram. Int..

[27] Hu Y., Li Z., Hu Z., Wang L., Ma R., Wang J. (2020). Engineering Hierarchical CoO Nanospheres Wrapped by Graphene via Controllable Sulfur Doping for Superior Li Ion Storage. Small.

[28] He S., Wu H., Li S., Liu K., Yang Y., Pan H., Yu X. (2024). Small but mighty: Empowering sodium/potassium-ion battery performance with S-doped SnO_2_ quantum dots embedded in N, S codoped carbon fiber network. Carbon Energy.

[29] Zheng J., Lin Y., Du C., Chen X., Li J., Zheng Y., Feng Q., Huang Z. (2023). Fluorine-doped MnO@fluorographene with high conductivity for improved capacity and prolonged cycling stability of lithium-ion anode. J. Alloys Compd..

[30] Zhong Y., Wang L., Yu Z., Li C., Wen Z., Xie J., Hu Y., Wang W., Hong G. (2021). Hierarchical Stratiform of a Fluorine-Doped NiO Prism as an Enhanced Anode for Lithium-Ion Storage. J. Phys. Chem. Lett..

[31] Lin Y., Zhong K., Zheng J., Liang M., Xu G., Feng Q., Li J., Huang Z. (2021). Fluorine-Doped GeO_2_@C Composite with Abundant Oxygen Vacancies for High-Capacity Lithium-Ion Batteries. ACS Appl. Energy Mater..

[32] He L., Sun W., Sun K., Mao Y., Deng T., Fang L., Wang Z., Chen S. (2022). Nitrogen and sulfur co-doped hierarchically mesoporous carbon derived from biomass as high-performance anode materials for superior sodium storage. J. Power Sources.

[33] Ruan J., Yuan T., Pang Y., Luo S., Peng C., Yang J., Zheng S. (2018). Nitrogen and sulfur dual-doped carbon films as flexible free-standing anodes for Li-ion and Na-ion batteries. Carbon.

[34] Wang K., Zhao K., Wang Y., Li H., Jiang H., Chen L. (2021). N, S co-doped carbon confined MnO/MnS heterostructures derived from a one-step pyrolysis of Mn-methionine frameworks for advanced lithium storage. J. Alloys Compd..

[35] Qi R., Jiang Q., Zhong M., Li W., Ren S., Wang Y., Feng M., Lu X. (2024). Manipulating d-band center of bimetallic Sn-alloy coupling with carbon nanofibers for high-performance electrocatalytic production of ammonia from nitrate. Chem. Eng. J..

[36] Zhang J., Lei Y., Zhou L., Chen X., Huang S., Liu L., Liu H., Dou S., Xu J. (2024). Ball-Milling Synthesis of Richly Oxygenated Graphene-Like Nanoplatelets from used Lithium Ion Batteries and Its Application for High Performance Sodium Ion Battery Anode. Adv. Funct. Mater..

[37] Ma S., Chen D., Wang W.-L. (2016). MnO nanoparticles embedded in a carbon matrix as high performance lithium-ion battery anodes: Preparation, microstructure and electrochemistry. Phys. Chem. Chem. Phys..

[38] Xiao Y., Wang X., Wang W., Zhao D., Cao M. (2014). Engineering Hybrid between MnO and N-Doped Carbon to Achieve Exceptionally High Capacity for Lithium-Ion Battery Anode. ACS Appl. Mater. Interfaces.

[39] Li Y., Zhao Y.-H., Zhao L.-L., Wang P.-F., Xie Y., Yi T.-F. (2024). Well-dispersed Sb particles embedded on N-doped carbon nanofibers toward high-performance Li-ion battery. Rare Met..

[40] Lin Z., Wu J., Ye Q., Chen Y., Jia H., Huang X., Ying S. (2024). Coral-like CoSe_2_@N-doped carbon with a high initial coulombic efficiency as advanced anode materials for Na-ion batteries. Dalton Trans..

[41] Liu Y.-P., Xu C.-X., Ren W.-Q., Hu L.-Y., Fu W.-B., Wang W., Yin H., He B.-H., Hou Z.-H., Chen L. (2023). Self-template synthesis of peapod-like MnO@N-doped hollow carbon nanotubes as an advanced anode for lithium-ion batteries. Rare Met..

[42] Cheng Y., Li S., Luo W., Li K., Yang X. (2024). N-Containing Porous Carbon-Based MnO Composites as Anode with High Capacity and Stability for Lithium-Ion Batteries. Molecules.

[43] Galakhov V.R., Demeter M., Bartkowski S., Neumann M., Ovechkina N.A., Kurmaev E.Z., Logachevskaya N.I., Mukovskii Y.M., Mitchell J., Ederer D.L. (2002). Mn3s exchange splitting in mixed-valence manganites. Phys. Rev. B.

[44] Zhang S., Xu Y., Cheng X., Gao S., Zhang X., Zhao H., Huo L. (2023). Coordination polymer-derived hierarchically structured MnO/NC composites as anode materials for high-performance lithium-ion batteries. J. Alloys Compd..

[45] Jia H., Wang Z., Li C., Si X., Zheng X., Cai Y., Lin J., Liang H., Qi J., Cao J. (2019). Designing oxygen bonding between reduced graphene oxide and multishelled Mn_3_O_4_ hollow spheres for enhanced performance of supercapacitors. J. Mater. Chem. A.

[46] Feng Y., Yang K., Jr R.L.S., Qi X. (2023). Metal sulfide enhanced metal–organic framework nanoarrays for electrocatalytic oxidation of 5-hydroxymethylfurfural to 2,5-furandicarboxylic acid. J. Mater. Chem. A.

[47] Shim K., Kim H.W., Park S., Seo K.-D., Kim C.-Y., Lee J.B., Bae J.S., Kim H.J. (2024). MnS/MnO-coated N, S-doped Carbon Anode obtained from a Mn(II)-coordinated Polymer for Long-cycle Life Li-ion Batteries. Mater. Adv..

[48] Peng C., Yue L., Cui Y., He X., Xu S., Guo C., Guo M., Chen H. (2023). Preparation of Cu_7.2_S_4_@N, S co-doped carbon honeycomb-like composite structure for high-rate and high-stability sodium-ion storage. J. Colloid Interf. Sci..

[49] Liu X., Li G., Wu J., Meng L., Zhang D., Zhang X., Li L. (2022). VN nanocrystals on N, S co-doped carbon framework: Topochemical self-nitridation and superior performance for lithium-ion battery. Electrochim. Acta.

[50] Zheng F., Deng Q., Zhong W., Ou X., Pan Q., Liu Y., Xiong X., Yang C., Chen Y., Liu M. (2018). Fluorine-Doped Carbon Surface Modification of Li-Rich Layered Oxide Composite Cathodes for High Performance Lithium-Ion Batteries. ACS Sustain. Chem. Eng..

[51] Wang P., Zhang K., Li H., Hu J., Zheng M. (2024). Enhanced Ion Transport Through Mesopores Engineered with Additional Adsorption of Layered Double Hydroxides Array in Alkaline Flow Batteries. Small.

[52] Lee C.R., Jang H.Y., Leem H.J., Lee M.A., Kim W., Kim J., Song J.H., Yu J., Mun J., Back S. (2024). Surface Work Function-Induced Thermally Vulnerable Solid Electrolyte Interphase Formation on the Negative Electrode for Lithium-Ion Batteries. Adv. Energy Mater..

[53] Zhang W., Li J., Zhang J., Sheng J., He T., Tian M., Zhao Y., Xie C., Mai L., Mu S. (2017). Top-Down Strategy to Synthesize Mesoporous Dual Carbon Armored MnO Nanoparticles for Lithium-Ion Battery Anodes. ACS Appl. Mater. Interfaces.

[54] Zhang D., Li G., Fan J., Li B., Li L. (2018). In Situ Synthesis of Mn_3_O_4_ Nanoparticles on Hollow Carbon Nanofiber as High-Performance Lithium-Ion Battery Anode. Chem. Eur. J..

[55] Wang R., Cao L., Li J., Xu Z., Huang J., Cui Y., Wang C. (2018). Design of dual-carbon modified MnO electrode improves adsorption and conversion reaction in Li-ion batteries. Ceram. Int..

[56] Zhang X., He X., Yin S., Cai W., Wang Q., Wu H., Wu K., Zhang Y. (2022). Rational Design of Space-Confined Mn-Based Heterostructures with Synergistic Interfacial Charge Transport and Structural Integrity for Lithium Storage. Inorg. Chem..

[57] Kim H., Choi W., Yoon J., Um J.H., Lee W., Kim J., Cabana J., Yoon W.-S. (2020). Exploring anomalous charge storage in anode materials for next-generation Li rechargeable batteries. Chem. Rev..

[58] Luo Q., Ming L., Zhang D., Wei C., Wu Z., Jiang Z., Liu C., Liu S., Cao K., Zhang L. (2023). Constructing Br-Doped Li_10_SnP_2_S_12_-Based All-Solid-State Batteries with Superior Performances. Energy Mater. Adv..

[59] Xiao P., Wang Z., Long K., Yang J., Liu X., Ling C., Chen L., Mei L. (2024). Stable cycling and low-temperature operation utilizing amorphous carbon-coated graphite anodes for lithium-ion batteries. RSC Adv..

[60] Ren J., Wang Z., Xu P., Wang C., Gao F., Zhao D., Liu S., Yang H., Wang D., Niu C. (2022). Porous Co_2_VO_4_ Nanodisk as a High-Energy and Fast-Charging Anode for Lithium-Ion Batteries. Nano-Micro Lett..

[61] Tian Q., Luo C., Yang D., Wang X., Chen M. (2024). A facile preparation route of MnO@C composite for high lithium storage anode. Chem. Phys. Lett..

[62] Yang L., Zhu X., Zhou Q., Qi C., Wang Q., Shi F., Zhu M., Chen G., Wang D., Liu X. (2024). Herringbone packed contorted aromatics with ordered three-dimensional channels as fast-charging and low-temperature lithium-ion battery anodes. J. Mater. Chem. A.

