# Supplementary material for: Concisely Constructing S, F Co-Modified MnO Nanoparticles Attached to S, N Co-Doped Carbon Skeleton as a High-Rate Performance Anode Material
Source: Molecules. 2024 Sep 11;29(18):4306. doi: 10.3390/molecules29184306 (PMC11434201; doi:10.3390/molecules29184306)
Supplement: Supplementary file 1 [file molecules-29-04306-s001.zip › molecules-3194763-supplementary.pdf]

Concisely constructing S, F Co-modified MnO Nanoparticles Attached to S, N Co-Doped Carbon Skeleton as a High-Rate Performance Anode Material

Dan Zhang [1,*], Chunyan Zhang [1], Zhe Huo[1], Jia Sun[1], Guangyin Liu[1], Xiaodi Liu[1] and Chuang Yu[2,*]

[1] College of Chemistry and Pharmaceutical Engineering, Nanyang Normal University, Nanyang 473061, PR China; zhangchunyanny@163.com (C.Z.); imkc62@163.com (Z.H.); sj18623839768@163.com (J.S.); liugy13@163.com (G.L.)

[2] State Key Laboratory of Advanced Electromagnetic Engineering and Technology, School of Electrical and Electronic Engineering, Huazhong University of Science and Technology, Wuhan 430074, PR China

* Correspondence: danzhangny@163.com (D.Z.); cyu2020@hust.edu.cn (C.Y.)

Electrochemical measurements

The electrochemical performance of the samples was tested using CR2025-type coin cell. The test electrodes were prepared by mixing the samples, carbon black, and binder (polyvinylidene fluoride (PVDF)) at a weight ratio of 7:2:1 in N-methyl-2-pyrrolidinone (NMP). The resulting slurries were coated onto a copper foil and then dried under vacuum at 80 ^{o}C for 12 h. Coin cells were assembled in an argon-filled glovebox (H_2O and O_2 < 0.1 ppm) using lithium foil as the counter and reference electrode, a polymer separator (Celgard 2500), and 1 M $LiPF_6$ in EC:DMC:DEC (1:1:1 in volume) as the electrolyte. The cells were tested using a LANHE Battery Test System in the potential range between 0.01 and 3 V at room temperature. Cyclic voltammetry (CV) was measured using an electrochemical workstation (CHI660C). Electrochemical impedance spectra (EIS) were characterized by the same instrument over a frequency range of 100 kHz to 0.01 Hz.

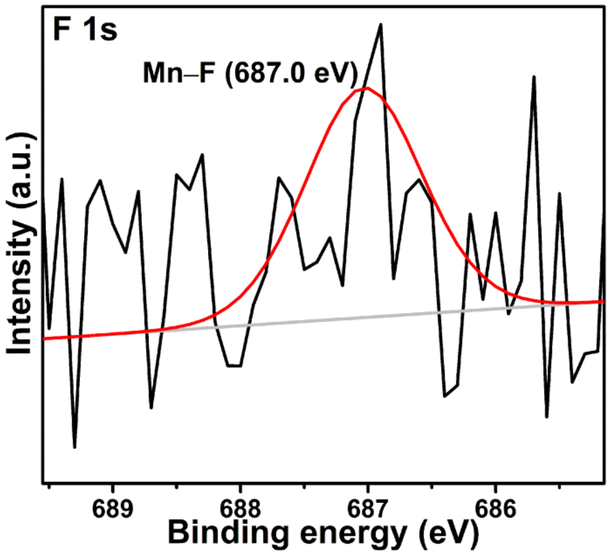

Figure S1 XPS spectrum of the F 1s region for SF-MnO/SNC composite.

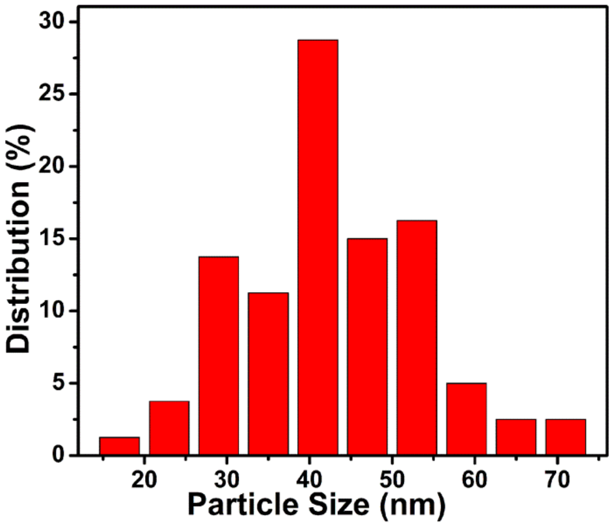

Figure S2 Nanoparticle size distribution diagram for SF-MnO/SNC composite.

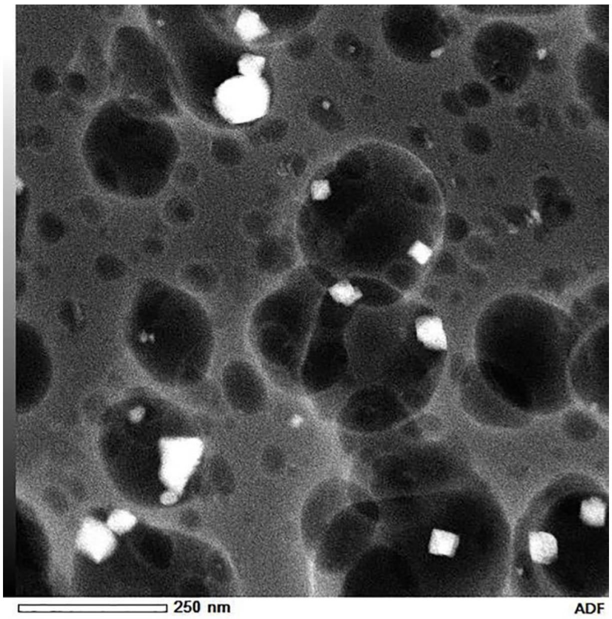

Figure S3 HAADF image of the elemental mapping for SF-MnO/SNC composite.

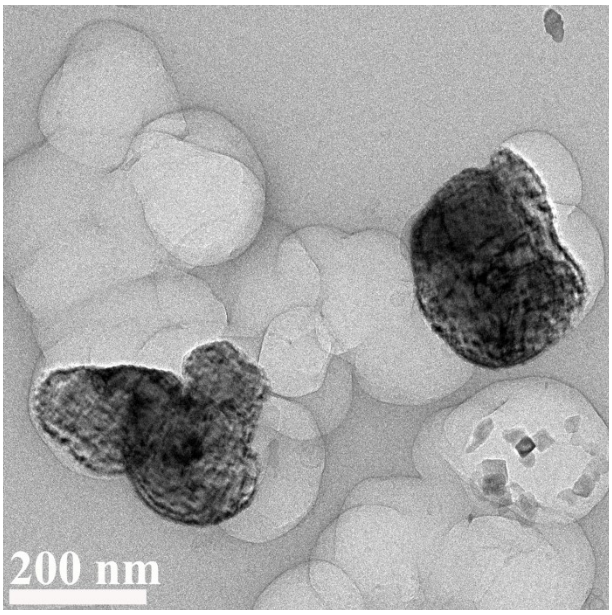

Figure S4 TEM image of S-MnO/SNC composite.

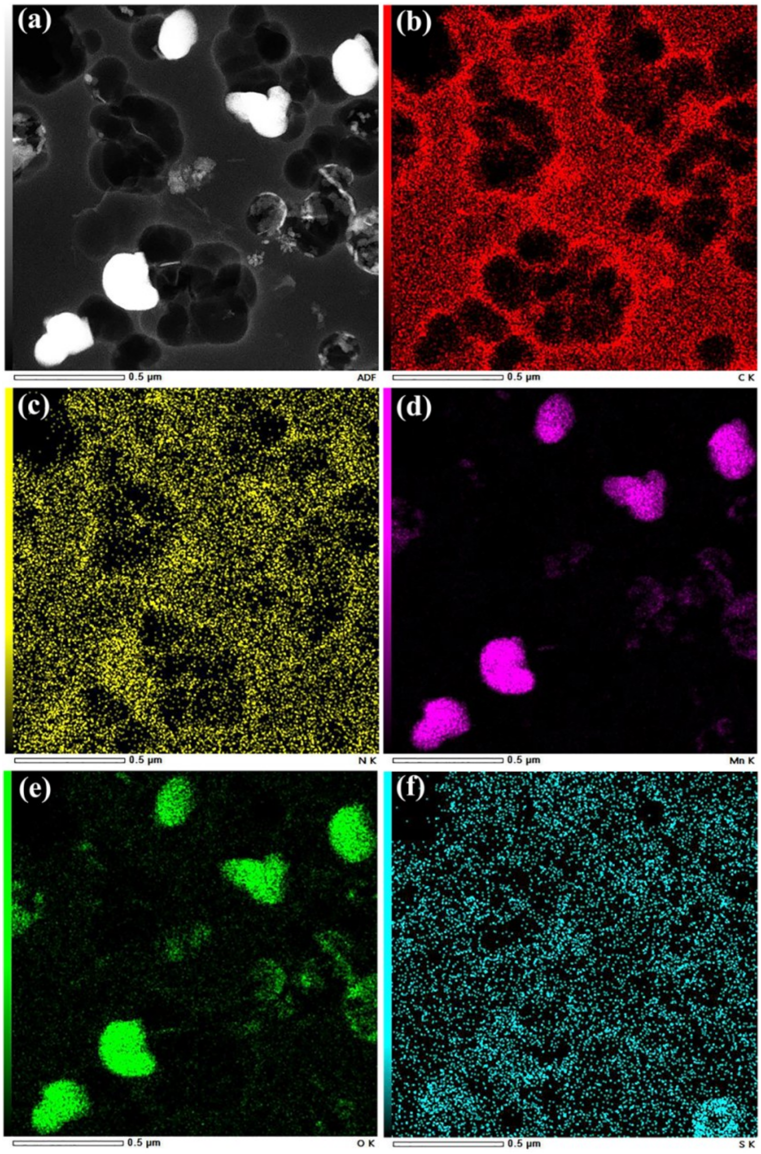

Figure S5 HAADF image of the elemental mapping (a) and EDS maps of elemental C (b), N (c), Mn (d), O (e) and S (f) for S-MnO/SNC composite.

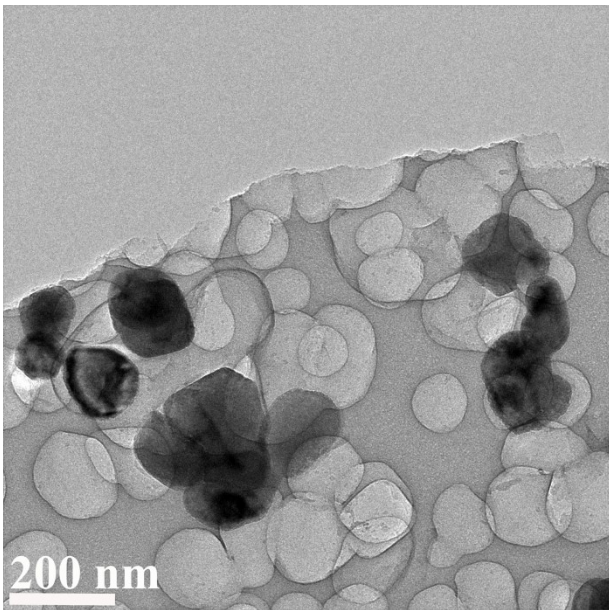

Figure S6 TEM image of F-MnO/NC composite.

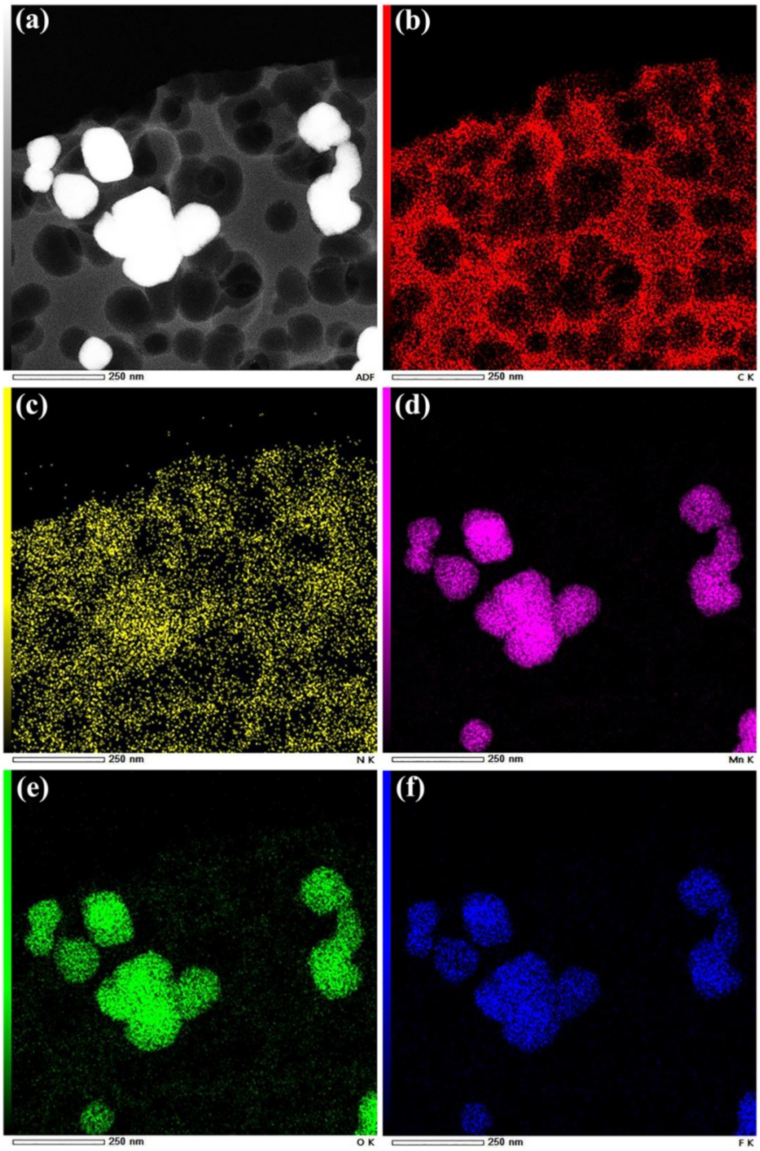

Figure S7 HAADF image of the elemental mapping (a) and EDS maps of elemental C (b), N (c), Mn (d), O (e) and F (f) for F-MnO/NC composite.

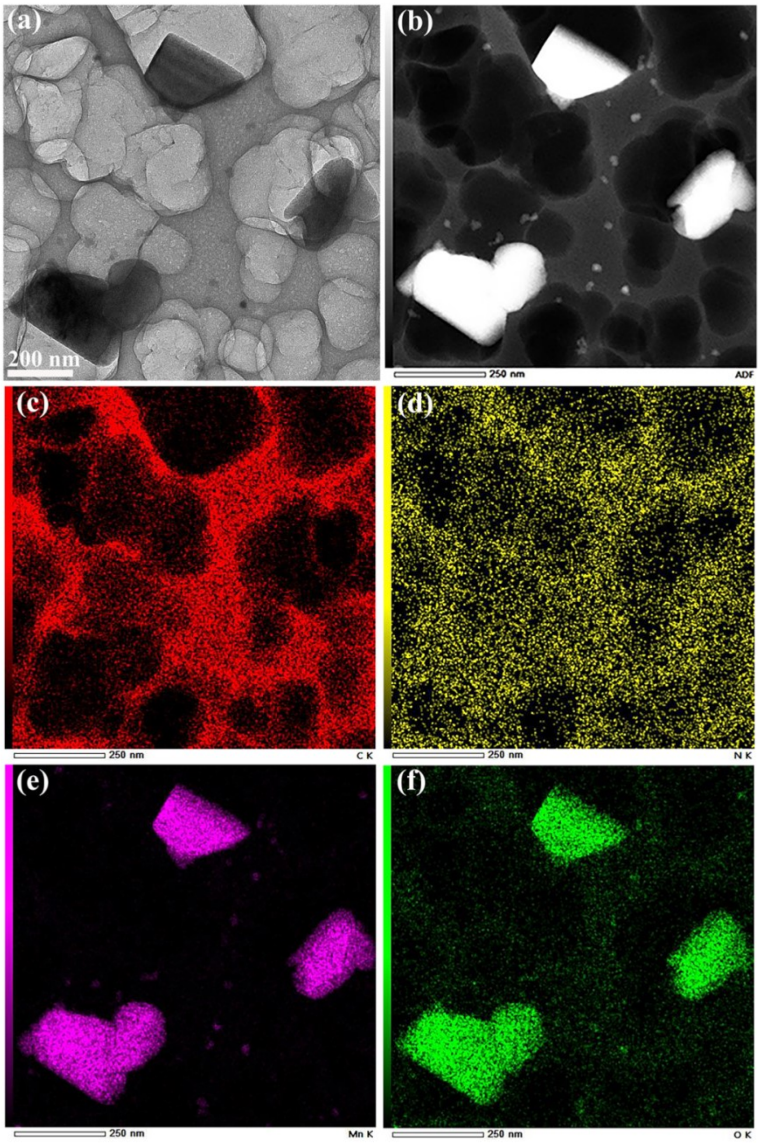

Figure S8 TEM image (a), HAADF image of the elemental mapping (b) and EDS maps of elemental C (c), N (d), Mn (e) and O (f) for MnO/NC composite.

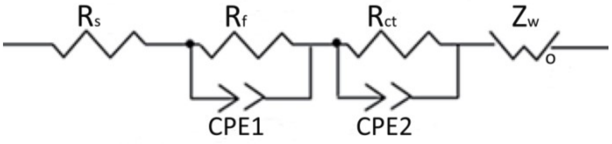

Figure S9 Equivalent circuit.

Table S1 EIS parameters for the SF-MnO/SNC, S-MnO/SNC, F-MnO/NC, and MnO/NC electrodes derived through the fitting of experimental data to an equivalent circuit model.

Sample	Cycle number	R_s/Ohm	R_f/Ohm	R_{ct}/Ohm
SF-MnO/SNC	after five cycles	5	27	35
S-MnO/SNC	after five cycles	9	41	54
F-MnO/NC	after five cycles	5	36	51
MnO/NC	after five cycles	5	62	70
